# Supplementary material for: Interaction between Lifestyle Changes and PNPLA3 Genotype in NAFLD Patients during the COVID-19 Lockdown
Source: Nutrients. 2022 Jan 27;14(3):556. doi: 10.3390/nu14030556 (PMC8838646; doi:10.3390/nu14030556)
Supplement: Supplementary file 1 [file nutrients-14-00556-s001.zip › nutrients-1500387-supplementary.pdf]

# SUPPLEMENTARY TABLES

**Supplementary Table S1.** Lifestyle and metabolic features before lockdown, according to age (< or > 67 years).

| Variable | Age < 67 ys (N= 222) | Age ⩾ 67 ys (N= 135) | P value |
|---|---|---|---|
| | *LIFESTYLE CHARACTERISITCS* | | |
| **Current smokers, n (%)** | 28 (13) | 10 (8) | 0.15 |
| **Light drinkers, n (%)** | 37 (17) | 42 (31) | **0.01** |
| **Alcohol intake, g/day** | 6 [6–12] | 12 [6–24] | **0.008** |
| **MD, n (%)** | 118 (56) | 68 (54) | 1.00 |
| **Regular PA, n (%)** | 82 (38) | 46 (35) | 0.57 |
| ➢ **Aerobic, n (%)** | 77 (36) | 46 (35) | |
| ➢ **Resistance, n (%)** | 5 (2) | 0 (0) | |
| **Inactive, n (%)** | 133 (62) | 87 (65) | |
| | *METABOLIC FEATURES* | | |
| **BMI, kg/m2** | 28.4 ± 4.4 | 28.3 ± 3.8 | 0.76 |
| ➢ **Overweight, n (%)** | 108 (50) | 71 (54) | 0.58 |
| ➢ **Obese, n (%)** | 66 (31) | 38 (29) | 0.72 |
| **Hypertension, n (%)** | 101 (45) | 97 (72) | **<0.001** |
| **T2DM, n (%)** | 40 (18) | 43 (32) | **0.004** |
| **Dyslipidemia, n (%)** | 104 (47) | 84 (62) | **0.008** |
| **Glycemia, mg/dL** | 102 ± 21 | 113 ± 26 | **<0.001** |
| **HDL cholesterol, mg/dL** | 49 ± 14 | 52 ± 12 | *0.05* |
| **LDL cholesterol, mg/dL** | 109 ± 34 | 90 ± 28 | **<0.001** |
| **Triglycerides, mg/dL** | 119 [92–174] | 110 [85–148] | 0.18 |
| | *LIVER DISEASE* | | |
| Increased ALT, n (%) | 60 (29) | 22 (17) | **0.01** |
| Increased AST, n (%) | 23 (11) | 9 (7) | 0.25 |
| Increased GGT, n (%) | 63 (32) | 33 (27) | 0.45 |
| **US steatosis, grade** | | | 0.06 |
| ➢ **1, n (%)** | 91 (41) | 67 (50) | |
| ➢ **2, n (%)** | 93 (42) | 53 (39) | |
| ➢ **3, n (%)** | 38 (17) | 15 (11) | |

§FIB4 not shown because age is part of the formula.

MD, Mediterranean diet. PA, physical activity. BMI, body mass index. T2DM, type 2 diabetes. HDL, high-density lipoprotein. LDL, low-density lipoprotein. ALT, alanine aminotransferase. AST, aspartate aminotransferase. GGT, gamma-glutamyltransferase. US, ultrasound. FIB-4, fibrosis 4 score.

**Supplementary Table S2.** Differences in lifestyle, metabolic features, and laboratory data before and after the lockdown, according to weight change and age (< or ≥ 67 years).

| | Patients without increased weight (n=184; n=102<67ys and n=82≥ 67 ys) | | | Patients with increased weight (n=170; n=117<67ys and n=53≥ 67 ys) | | |
|---|---|---|---|---|---|---|
| **Variable** | **Before lockdown** | **After lockdown** | **P value** | Before lockdown | After lockdown | P value |
| *LIFESTYLE CHARACTERISTICS* | | | | | | |
| **Current smokers, n (%)** | | | | | | |
| ➢ **Age < 67 ys** | 13 (13) | 10 (10) | 0.08 | 15 (14) | 15 (14) | 1.00 |
| ➢ **Age ⩾ 67 ys** | 7 (9) | 4 (5) | 0.08 | 3 (6) | 1 (2) | 0.16 |
| **Light drinkers, n (%)** | | | | | | |
| ➢ **Age < 67 ys** | 20 (18) | 22 (14) | 0.83 | 22 (16) | 23 (14) | 0.49 |
| ➢ **Age ⩾ 67 ys** | 34 (38) | 37 (34) | 0.17 | 15 (24) | 17 (22) | 0.57 |
| **MD, n (%)** | | | | | | |
| ➢ **Age < 67 ys** | 53 (54) | 62 (67) | *0.05* | 64 (58) | 49 (44) | **0.001** |
| ➢ **Age ⩾ 67 ys** | 38 (50) | 38 (54) | 0.83 | 30 (65) | 23 (49) | **0.03** |
| **Regular PA, n (%)** | | | | | | |
| ➢ **Age < 67 ys** | 36 (36) | 40 (41) | 0.41 | 45 (40) | 26 (23) | **0.002** |
| ➢ **Age ⩾ 67 ys** | 31 (39) | 26 (32) | 0.25 | 15 (28) | 17 (32) | 0.59 |
| *METABOLIC FEATURES* | | | | | | |
| **T2DM, n (%)** | | | | | | |
| ➢ **Age < 67 ys** | 17 (17) | 17 (17) | 1.00 | 23 (20) | 23 (20) | 1.00 |
| ➢ **Age ⩾ 67 ys** | 24 (29) | 24 (29) | 1.00 | 19 (36) | 19 (36) | 1.00 |
| **Hypertension, n (%)** | | | | | | |
| ➢ **Age < 67 ys** | 42 (41) | 44 (43) | 0.16 | 57 (49) | 57 (49) | 1.00 |
| ➢ **Age ⩾ 67 ys** | 60 (73) | 61 (75) | 0.32 | 37 (70) | 37 (70) | 1.00 |
| **Dislipidemia, n (%)** | | | | | | |
| ➢ **Age < 67 ys** | 46 (45) | 48 (47) | 0.16 | 56 (49) | 60 (52) | 0.08 |
| ➢ **Age ⩾ 67 ys** | 50 (61) | 50 (61) | 1.00 | 34 (64) | 35 (66) | 0.32 |
| **Glycemia** | | | | | | |
| ➢ **Age < 67 ys** | 101 ± 18 | 99 ± 16 | 0.16 | 103 ± 23 | 109 ± 32 | **0.004** |
| ➢ **Age ⩾ 67 ys** | 111 ± 25 | 109 ± 24 | 0.43 | 115 ± 27 | 117 ± 31 | 0.18 |
| **HDL cholesterol** | | | | | | |
| ➢ **Age < 67 ys** | 46 ± 12 | 47 ± 13 | 0.56 | 51 ± 14 | 51 ± 16 | 0.17 |
| ➢ **Age ⩾ 67 ys** | 52 ± 12 | 51 ± 13 | 0.53 | 53 ± 12 | 53 ± 14 | 0.98 |
| **LDL cholesterol** | | | | | | |
| ➢ **Age < 67 ys** | 114 ± 35 | 114 ± 34 | 0.65 | 106 ± 33 | 112 ± 34 | 0.24 |
| ➢ **Age ⩾ 67 ys** | 91 ± 32 | 93 ± 33 | 0.74 | 88 ± 22 | 89 ± 29 | 0.49 |
| **Triglycerides** | | | | | | |
| ➢ **Age < 67 ys** | 129 [98–187] | 128 [97–173] | 0.11 | 114 [89–163] | 116 [96–172] | 0.32 |
| ➢ **Age ⩾ 67 ys** | 112 [86–147] | 119 [91–134] | 0.38 | 110 [81–159] | 123 [94–153] | 0.82 |
| *LIVER DISEASE* | | | | | | |
| **Increased ALT, n (%)** | | | | | | |
| ➢ **Age < 67 ys** | 32 (33) | 26 (28) | 0.25 | 28 (25) | 43 (38) | **0.005** |
| ➢ **Age ⩾ 67 ys** | 15 (19) | 12 (15) | 0.18 | 7 (13) | 6 (11) | 0.65 |
| **Increased AST, n (%)** | | | | | | |
| ➢ **Age < 67 ys** | 13 (13) | 12 (13) | 0.53 | 10 (9) | 17 (15) | **0.05** |
| ➢ **Age ⩾ 67 ys** | 5 (6) | 7 (9) | 0.16 | 4 (8) | 3 (6) | 0.65 |
| **Increased GGT, n (%)** | | | | | | |
| ➢ **Age < 67 ys** | 32 (35) | 29 (32) | 0.71 | 30 (28) | 42 (38) | **0.02** |
| ➢ **Age ⩾ 67 ys** | 24 (33) | 23 (31) | 0.71 | 9 (18) | 13 (27) | 0.26 |
| **US steatosis** | | | | | | |
| ➢ **Age < 67 ys** | | | 0.13 | | | 0.09 |

| | | | | | | |
|---|---|---|---|---|---|---|
| • **0-1, n (%)** | 52 (51) | 44 (43) | | 41 (35) | 33 (28) | |
| • **2-3, n (%)** | 50 (49) | 58 (57) | | 76 (65) | 84 (72) | |
| ➢ **Age ⩾ 67 ys** | | | | | | |
| • **0-1, n (%)** | | | | | | |
| • **2-3, n (%)** | 38 (46) | 38 (46) | 1.00 | 29 (54) | 19 (36) | **0.007** |
| | 44 (54) | 44 (54) | | 24 (46) | 34 (64) | |
| **FIB-4** | | | | | | |
| ➢ **Age < 67 ys** | 1.06 [0.74-1.44] | 1.11 [0.79-1.39] | 0.93 | 1.08 [0.84-1.37] | 1.13 [0.83-1.50] | 0.07 |
| ➢ **Age ⩾ 67 ys** | 1.62 [1.26-2.06] | 1.75 [1.27-2.13] | *0.05* | 1.58 [1.34-2.23] | 1.52 [1.29-2.24] | 0.98 |

MD, Mediterranean diet. PA, physical activity. T2DM, type 2 diabetes. HDL, high-density lipoprotein. LDL, low-density lipoprotein. ALT, alanine aminotransferase. AST, aspartate aminotransferase. GGT, gamma-glutamyltransferase. US, ultrasound. FIB-4, fibrosis 4 score.
